# Exploration of Workplace Bullying among Nurses: A Focus on Clinical Settings

**DOI:** 10.3390/healthcare12171706

**Published:** 2024-08-26

**Authors:** Manal F. Alharbi, Sami M. Alotebe, Turki M. Alotaibi, Nawal A. Sindi, Dalal N. Alrashidi, Hala K. Alanazi

**Affiliations:** 1 Maternal & Child Health Nursing Department, College of Nursing, King Saud University, Riyadh 11451, Saudi Arabia; 2 Department of Nursing, College of Applied Medical Sciences, University of Hafr Al-Batin, Hafr Al-Batin 39524, Saudi Arabia; smalotebe@uhb.edu.sa; 3 Nursing Administration, Dawadmi General Hospital, Ministry of Health, Al Dawadmi 17463, Saudi Arabia; tumoalotaibi@moh.gov.sa; 4 General Administration of Home Health Care, Ministry of Health, Riyadh 12613, Saudi Arabia; nawals@moh.gov.sa; 5 Alyamamh Hospital, Ministry of Health, Riyadh 12613, Saudi Arabia; dnalrashidi@moh.gov.sa; 6 Nursing Office, Prince Mohammed Bin Abdulaziz Hospital, Ministry of Health, Riyadh 14214, Saudi Arabia; halanazi71@moh.gov.sa

**Keywords:** workplace, violence, nursing, practice, environmental health, clinical settings

## Abstract

Purpose: Healthcare practitioners in Saudi Arabia sometimes experience workplace bullying (WPB). However, more research on this issue must be carried out in the country. This study aimed to investigate the prevalence of WPB and how individual factors among nurses contribute to different experiences of WPB in clinical settings. Methods: This cross-sectional quantitative study occurred among registered nurses in Riyadh, Saudi Arabia. A self-administered questionnaire was used, and nurses filled it out via a Google survey that included sociodemographic details such as age, gender, education, and the WBS to gauge the prevalence of workplace bullying in hospital settings. Results: Of 416 nurses, 58.7% were aged between 31 and 40, and 76.9% were women. The prevalence of WPB was found to be 54.8%. WPB was higher among middle-aged nurses, men, charge/managerial nurses, nurses with higher education, those with 3 to 6 years of experience, and those working in specialty units. Conclusions: More than half of the nurses had experienced WPB at some point. Middle-aged nurses, especially men with higher education and more experience in specialty units, were the most common victims of WPB compared to other registered nurses.

## 1. Introduction

“Workplace bullying” (WPB) refers to the repeated and unreasonable abuse of an individual or group. Sexual, physical, or verbal harassment is a form of WPB [1]. According to the operational definition, the circumstances in which one or more people are repeatedly subjected to unpleasant activities from others over an extended period are defined as bullying. Bullying is abusive conduct that includes many different forms, such as abusive words and phrases, behaviors that threaten, intimidate, or humiliate (including nonverbal threats), and the interruption of work—sabotage—preventing work completion [2]. Workplace bullying is known as lateral violence or horizontal violence and involves repeated ill-treatment of a person or persons [2]. However, there is no formally accepted standard definition [3]. Moreover, the phrase “workplace violence” is sometimes used to describe bullying; however, it most frequently relates to physical violence or threats of damage [4]. The term workplace incivility is often used with WPB, horizontal/lateral violence, psychosocial harassment, and intimidating behavior interchangeably [5]. Approximately 44 percent of nursing staff in healthcare settings have been bullied [2]. The prevalence of bullying may vary significantly between nations and even within a single nation. For instance, 11.3% of healthcare workers report being bullied at work [6]. However, these findings may depend on the survey and assessment techniques.

**Figure 1:**
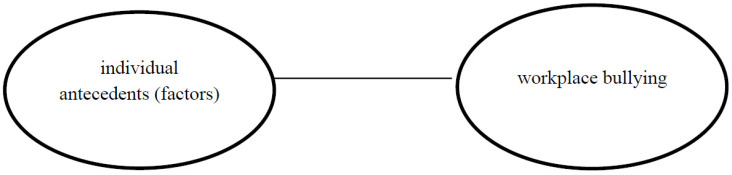
Study framework. Note. This model represents relationships between study variables of the individual factors and workplace. bullying (Anjum et al., 2019 [14]).

Bullying has become a severe and expanding issue in healthcare settings, affecting a sizeable portion of healthcare professionals. Understanding the elements that contribute to the origin and growth of bullying is crucial because of its detrimental effects on the mental health and well-being of employees and, consequently, on the performance of the organization [6]. WPB has numerous and well-documented causes. Some causes are attributed to individual factors, such as a lack of professional experience, role conflicts, low self-confidence, and dissatisfied employees. Environmental factors, such as work overload, inadequate managerial support, and poor communication, are other causes of WPB [7]. However, victims of bullying experience a decrease in job satisfaction, work performance, motivation, and productivity. Their social ties, both inside and outside the institution, are adversely affected [8]. WPB bullying among nurses is a recurrent problem and most common among nurses in healthcare settings. In response, organizations such as the American Nursing Association (ANA) have created statements on workplace bullying, violence, and incivility [9]. This phenomenon may be attributed to long working hours, high workloads, and direct contact with patients and their relatives [10]. According to Ahmed, ref. [11] WPB was reported by 57.3% of individuals in the previous year, posing a concern. WPB significantly impacts nursing practices, employee retention, and job satisfaction. It disrupts the office environment, damages managerial credibility, and endangers patient safety [12]. WPB dramatically impacts nurses’ emotional well-being and productivity in healthcare settings. One study that examined WPB in the Middle East reported that bullying was highly prevalent, particularly in emergency departments. However, significant gaps exist in the research on other departments and units [13]. Al Omar [12] argued that most healthcare providers in Saudi Arabia (SA) consider WPB a concern; it negatively influences the quality of care and patient safety, thus necessitating similar studies to gather more information about the influence of WPB on patient safety in SA. Al-Surimi [1] investigated WPB prevalence among Saudi healthcare professionals and demonstrated that 63% of the participants reported bullying from their patients. A nursing study conducted in a tertiary hospital in Riyadh reported that 33.4% of the participants reported being victims or witnesses of WPB [7]. Furthermore, approximately one-third of the respondents planned to leave their current jobs. However, few studies have been conducted on WPB among nurses in Saudi Arabia. It is crucial to establish a safer and more supportive work environment for nurses. By gaining insight into the current status and prevalence of workplace bullying, we can take the necessary steps to address this issue within the Saudi Arabian nursing context. Moreover, identifying the contributing factors (antecedents) can inform the development of better policies and training programs. When staff members are aware of expected behavior and norms, bullying is likely to decrease. Proactively preventing bullying can enhance overall workplace morale and productivity. When individuals feel valued and secure, they are more likely to find fulfillment in their roles and perform at higher levels of quality. Understanding and addressing workplace bullying in healthcare facilities will safeguard employees and cultivate a healthier, more conducive work environment where everyone can flourish. Therefore, the theoretical framework provides the structure and rationale for this research study, helping to identify relationship patterns among variables. Referring to Figure 1, the study model comprises two constructs, individual and organizational factors, both related to workplace bullying. The examination of workplace bullying among nurses involves the use of the workplace bullying scale (WBS) to assess the level of workplace bullying (dependent variable) based on individual and organizational factors (independent variables) among nurses. The scientific hypotheses (H1) represent the expected relationships between the dependent variable and independent variables in this study. All research hypotheses are inductive and direct. Initially, the focus is on the individual factors related to workplace bullying among nurses, followed by exploring the organizational factors linked to this issue. Therefore, we aimed to examine WPB prevalence among registered nurses in clinical settings across Riyadh. Moreover, we intended to investigate different experiences of WPB attributed to dissimilar individual factors among the nurses in clinical settings.

## 2. Materials and Methods

### 2.1. Study Design

In this quantitative cross-sectional study, we assessed the respondents in selected healthcare settings from February to May 2023. We adopted a descriptive correlational design to determine whether changes in one variable were associated with changes in another and to examine the relationships between and among the variables. This approach helped us explore the current situation—a veritable data snapshot—in an inexpensive and rapidly implementable manner.

### 2.2. Sample and Setting

The sample was selected using randomized multistage cluster sampling as a research method used to collect data from a large population. Subsequently, we selected a cross-section of the population working across five randomly selected hospitals in the Riyadh region. Following that, we specified the population for our study and partitioned it into smaller clusters within different cities, namely, Riyadh, Dawadmi, Shaqra, and Quayah. Subsequently, we randomly selected five hospitals (Prince Mohammad bin Abdulaziz Hospital, Alyamama Hospital, Dawadmi Hospital, Shaqra Hospital, and Quayah Hospital). We then took samples from within these clusters at the selected hospitals, forming smaller groups for our research. This approach helps to save time and cut costs when dealing with large populations while yielding valuable and representative data. Then, we used an identical form of sampling to include all specialty and general wards in the selected hospitals. Subsequently, specific nurses were selected through systematic random selection from hospitals and wards chosen randomly. The inclusion criteria for the selected participants were (1) nurses who held their position for at least three months, (2) currently working in hospitals or units in Riyadh, and (3) had a license through the Saudi Commission for Health Specialties. Staff with <3 months of experience were excluded. The Raosoft website (http://www.raosoft.com/samplesize.html) (accessed on 20 October 2022) was used to determine the appropriate size for the selected sample. To obtain a low margin of error (5%) and a high confidence level (95%), the website determined the sample size as 377. Eventually, we recruited a sample size of N = 377 + 10%, which would allow for missing data.

### 2.3. Measurements

The first part of the questionnaire consisted of questions about the respondents’ demographics, such as age, marital status, sex, and nationality. The second part consisted of individual factors, such as education level, job position, length of service, and work area. The final part, composed of a 21-item Work Bullying Scale (WBS), was developed to assess workplace bullying in workplace environments [14]. Respondents used a five-point Likert scale to report the frequency of bullying they encountered, with response options ranging from “never” (scored as 1) to “daily” (scored as 5). The tool has demonstrated strong validity and reliability, with a Cronbach’s alpha of 0.91 when used to evaluate workplace bullying (WPB) in nursing [14]. Specifically, Cronbach’s alpha scores for person-related and work-related bullying were 0.87 and 0.77, respectively [14]. The work-related bullying items involved threats to one’s professional life and comprised 10 specific items from the WBS (items 5, 6, 7, 12, 13, 15, 16, 17, 18, and 19). On the other hand, the person-related bullying items included attacks on one’s personality and personal life and were made up of 11 distinct items from the WBS (items 1, 2, 3, 4, 8, 9, 10, 11, 14, 20, and 21) [14].

### 2.4. Data Collection

In healthcare and nursing research, surveys are important data collection methods that can be administered differently [15]. We used an electronic survey involving web-based questionnaires. One advantage of this method is its low cost. Scott [16] mentioned that online surveys save printing costs because the data are entered exclusively online. Questionnaires were distributed using Google, an online website. However, we adopted different strategies to increase the response rates. For example, emails containing study details and a link to the online questionnaire on the study website were sent to the participants. This email also provided an estimate of the time required to complete the questionnaire [15] to improve the response rates. Moreover, automatic email reminders were sent to the participants, particularly to alert those who failed to respond at any point during data collection. According to McPeake [15] and Bryman [17], follow-ups and reminders are two essential strategies for maximizing responses to an online survey. The questionnaire was designed to motivate the participants to complete it. Bryman [17] reported that an attractive questionnaire design can improve response rates. However, we obtained a minimum response rate of 50%.

### 2.5. Data Analysis

The questionnaire criteria were analyzed based on previous research. WPB prevalence was identified by classifying the individuals who experienced at least two negative behaviors weekly or more often for ≥6 months [18,19]. Categorical variables are presented as numbers and percentages. Continuous variables are described as means and standard deviations. The WBS scores were compared with the sociodemographic characteristics of the nurses using the Mann–Whitney *Z*-test and Kruskal–Wallis *H*-test. Statistical collinearity was measured using the Shapiro–Wilk and Kolmogorov–Smirnov tests. Work- and person-related bullying, as did the overall WBS scores, followed non-normal distributions. Therefore, we performed non-parametric tests. Statistical significance was set at *p* < 0.05. All statistical analyses were performed using the Statistical Packages for Software Sciences version 21 (IBM Corporation, Armonk, NY, USA), with no missing data.

### 2.6. Ethical Consideration

Before beginning this research, we obtained approval from the institutional review board of the Ministry of Health (23-2E). All accepted practices were followed, including informed consent, voluntary participation, confidentiality, anonymity, privacy, beneficence, and nonmaleficence. We provided all the participants with a letter explaining the aim of the research without access to their personal information.

## 3. Results

In total, 416 nurses were enrolled in this study. Table 1 summarizes their sociodemographic characteristics. Approximately 58.7% of the nurses were aged between 31 and 40, with 75.9% being women. Married nurses accounted for 61.8% of the participants. Regarding professional ranking, 61.5% of the participants were staff nurses. Most nurses had a bachelor’s degree or higher (89.2%). Half the nurses (50.5%) had >6 years of experience, and 51.4% worked in a specialty unit. In addition, 30% worked at Prince Mohammed Bin Abdulaziz Hospital.

The mean scores for work- and person-related bullying were 22.0 and 24.8, respectively (Table 2). The overall mean score for WPB was 46.8 (SD = 20.2). Accordingly, 54.8% of the participants reported WPB, whereas 45.2% did not report bullying.

While measuring the association between WPB and their sociodemographic characteristics (Table 3), the nurses aged between 31 and 40 years demonstrated higher work-related bullying (*H* = 8.514; *p* = 0.014), person-related bullying (*H* = 8.518; *p* = 0.014), and overall WBS (*H* = 8.813; *p* = 0.012) scores. Men reported higher work-related bullying (Z = 2.473; *p* = 0.013), person-related bullying (*Z* = 2.207; *p* = 0.027), and overall WBS (*Z* = 2.412; *p* = 0.016) scores. The charge and managerial nurses demonstrated higher work-related bullying (*Z* = 3.094; *p* = 0.002), person-related bullying (*Z* = 2.647; *p* = 0.008), and overall WBS (*Z* = 2.979; *p* = 0.003) scores. Nurses with better education reported higher work-related bullying (*Z* = 4.067; *p* < 0.001), person-related bullying (Z = 3.928; *p* < 0.001), and overall WBS (*Z* = 4.128; *p* < 0.001) scores. Furthermore, nurses with 3 to 6 years of experience demonstrated higher work-related bullying (*H* = 33.172; *p* < 0.001), person-related bullying (*H* = 28.722; *p* < 0.001), and overall WBS (*H* = 32.539; *p* < 0.001) scores. Nurses in specialty units reported higher work-related bullying (*Z* = 2.171; *p* = 0.030) and overall WBS (*Z* = 2.074; *p* = 0.038) scores. Finally, nurses working at Prince Mohammed Bin Abdulaziz Hospital were more likely to report higher person-related bullying (*H* = 18.684; *p* = 0.001) and overall WBS (*H* = 22.612; *p* < 0.001) scores, whereas those working at Quaya Hospital were more likely to report higher work-related bullying (*H* = 26.233; *p* < 0.001) scores.

## 4. Discussion

We examined WPB prevalence and its experiences based on different individual factors among nurses in clinical settings. WPB occurs in varying degrees in hospital settings, which aligns with our results. According to the WBS criteria, WPB prevalence was 54.8%. The overall mean WBS was 46.2 (SD, 20.2), consistent with that reported by Chowdhury [20]. Approximately 61.8% of the participants experienced WPB, including 54.2% experiencing burnout and 16.3% complaining of “intermediate to high” workplace violence in the previous year. This finding has confirmed that 63.7% of nurses experienced WPB, and the most common perpetrators were the patients (36.1%) or their families and relatives (36.1%) [1,12]. However, a relatively higher prevalence of bullying has been reported in Jordan, ref. [13] accounting for 90% of the reports among emergency department nurses, which exerts a significant effect on the nurses’ perception of productivity and the quality of care provided. Our results considerably agree with the notion of the presence of WPB. Hence, anti-bullying measures must be strictly implemented to reduce WPB in healthcare settings.

This study makes significant contributions to the existing literature. Moreover, our results will be valuable for nurses in SA worldwide. They will help improve interventions and create best practice standards in care settings. Our findings will support the implementation of the health chapter in SA’s Vision 2030, considering the varying degrees of concern and worry about workplace bullying (WPB) among healthcare workers [1,12]. WPB can often cause workplace stress and burnout, thus warranting a need to understand how it affects the workplace climate in different and specific nursing settings. The possibility of workplace stress and burnout caused by WPB necessitates effective measures and interventions to address and mitigate these issues. Considering the shortage of quality healthcare workers and nurses worldwide, newly qualified nurses frequently face incivility that may negatively impact their self-esteem and confidence, ultimately impacting their workforce participation decisions and what they do at work [21]. The study model has two constructs (Figure 1). In other words, these two factors were related to WPB. We used the workplace bullying scale (WBS) to examine the WPB rate (dependent variable) among nurses, depending on individual factors (independent variable). Regarding the scientific hypotheses, H1 is the expectation of a relationship between the dependent and independent variables. However, all research hypotheses are inductive and direct. First, the individual factors were related to WPB among nurses.

The study variables were divided into dependent and independent variables, defined conceptually and operationally, to explain the process of measuring and collecting the information needed in this study. In addition, WPB refers to purposeful breaches of an individual’s dignity, rights, and integrity [14].

Nurses working in speciality units may be at the receiving end of WPB [11,13]. Similarly, nurses working in speciality units were more prone to WPB than others. However, in Spain, six healthcare workers are bullied if they work on a shift schedule, perform monotonous and rotating tasks, experience work stress, enjoy marginal satisfaction with their working conditions, or seek opportunities for promotion in their organizations. These findings may be consistent with the study by Hosier, [22], who identified the significant predictors of WPB, such as personality, working hours per week, work environment, and emotional stability. WPB substantially affects nurses’ job satisfaction, stress, and turnover intention. However, it does not influence work productivity or absenteeism.

Moreover, significant risk factors for WBP regarding the sociodemographic variables included the middle-aged (31 to 40), men, a charge nurse or manager, higher education, 3 to 6 years of experience, and employment at the Prince Mohammed Bin Abdulaziz Hospital. Because 30% of the sample was recruited from this hospital, our results should be interpreted cautiously. Similarly, a study in Malaysia reported that having been in the workplace for less than ten years and not contributing to direct patient care were statistically significant factors associated with workplace bullying [23].

In a study conducted in Riyadh, ref. [1] WPB was more prevalent among younger nurses but less prevalent among higher-educated nurses. Among the physicians, a higher prevalence was observed in women and those with higher education. In China, ref. [24] WPB prevalence varies significantly according to age, department, years of experience, and hospital level. However, a study in Riyadh [7] reported an inverse correlation between age and WPB, in contrast to a positive association with turnover intention.

Al Muharraq [7] demonstrated that work-related bullying behaviors formed the most common bullying acts (34.5%), followed by personal bullying (31.1%). Physical intimidation accounted for 25.6% of work-related bullying behaviors. Of all participants, 31.7% exhibited a high turnover intention. Similarly, Baburajan [25] documented that 80%, 60%, and 21.3% of the staff nurses who experienced WPB reported work-related bullying, person-related bullying, and physical intimidation, respectively. In Egypt, 60.6% of the nurses reported WPB perpetrated by patients and their relatives, whereas their supervisors and managers accounted for 33.3% [26]. In our study, the mean score for person-related bullying (24.8) was higher than that of work-related bullying (22.0). This finding suggests that person-related bullying occurs more frequently than work-related bullying. Furthermore, significant predictors of increased work-related and person-related bullying were identified as middle-aged groups, men, charge/managerial nurses, a bachelor’s degree or higher, and 3 to 6 years of working experience; these factors may be due to cultural perspectives, and the individual may choose to remain silent in response to workplace bullying. A study conducted in an Indian context revealed a positive correlation between workplace bullying and defensive, relational, and ineffectual silence [27]. The study also found that the mediation of the bullying–silence relationship was through psychological contract violation (PCV) and that workplace friendship moderated this mediation pathway. This study is particularly interesting as it is one of the few to explore passive coping strategies in response to workplace mistreatment and the impact of bullying on outcomes in an Indian context [27]. The findings suggest that implementing an anti-bullying policy and fostering management support may empower employees to address bullying by speaking out against it.

### 4.1. Implications

Healthcare facilities can implement various strategies to address workplace bullying. These include developing clear policies and strictly enforcing comprehensive anti-bullying measures. Providing ongoing educational programs, such as regular training sessions on professional conduct and conflict resolution, is essential. Additionally, creating secure channels for reporting bullying incidents is crucial to empower nurses to report without fear of retaliation. Offering counselling and support services for affected nurses is equally essential. Management’s active involvement ensures that bullying reports are taken seriously and addressed promptly. Fostering a positive work environment that emphasizes mutual respect, teamwork, and open communication is also crucial. By proactively addressing workplace bullying, healthcare institutions can foster a supportive environment for nurses, ultimately leading to improved patient care and operational efficiency within the healthcare system.

### 4.2. Limitations

The study had some limitations. It was limited to government-run medical facilities in a specific region, potentially limiting its broader relevance. Data collection relied on self-administered surveys, which may have introduced bias as participants might have hesitated to provide entirely honest responses. In addition, the cross-sectional research design lacks the ability to establish cause and effect as it only offers a snapshot of a single moment, and the composition of the study sample significantly impacts its findings. Additionally, the study needed more detailed information about the experiences of nursing supervisors. Despite these constraints, the research provides valuable insights into workplace bullying in the Saudi Arabian nursing sector. Future studies with a broader scope and more effective data collection techniques could significantly enhance our understanding of this issue. These improvements would contribute to a more comprehensive understanding of the problem and pave the way for more effective interventions.

## 5. Conclusions

The study revealed a high prevalence of workplace bullying (WPB) in hospital settings, particularly among middle-aged nurses, male nurses, charge/managerial nurses, those with higher education, those with 3 to 6 years of experience, and nurses working in specialty units. This study underscores the need for further investigation and interventions to combat workplace bullying in Saudi Arabia. Understanding and addressing this issue can enable hospital administrators to create a more supportive work environment. By identifying the most affected areas and individuals, they can implement training programs, foster teamwork, and promote open communication to reduce bullying, leading to a more contained and efficient staff. This, in turn, can enhance patient care and cultivate a more positive hospital atmosphere.

## Figures and Tables

**Table 1:** Sociodemographic characteristics of the nurses (n = 416).

Study Variables	n (%)
Age group	
20–30 years	106 (25.5%)
31–40 years	244 (58.7%)
41–50 years	57 (13.7%)
>50 years	09 (02.2%)
Sex	
Male	96 (23.1%)
Female	320 (76.9%)
Marital status	
Single	135 (32.5%)
Married	257 (61.8%)
Divorced or widowed	24 (05.8%)
Professional rank	
Staff Nurse	256 (61.5%)
Charge nurse	160 (38.5%)
Educational level	
Diploma	45 (10.8%)
Bachelor or higher	371 (89.2%)
Years of experience	
<3 years	67 (16.1%)
3–6 years	139 (33.4%)
>6 years	210 (50.5%)
Work area	
General unit	202 (48.6%)
Specialty unit	214 (51.4%)
Hospital	
Alyamama Hospital	84 (20.2%)
Dawadmi Hospital	90 (21.6%)
Prince Mohammad Bin Abdulaziz Hospital	125 (30.0%)
Quayah Hospital	62 (14.9%)
Shaqra Hospital	55 (13.2%)

**Table 2:** Prevalence of workplace bullying according to workplace bullying scale (WBS) (n = 416).

Variables	Mean ± SD
Work-related bullying score	22.0 ± 10.2
Person-related bullying score	24.8 ± 10.4
Overall WBS score	46.8 ± 20.2
Prevalence of WBS	n (%)
Bullied	228 (54.8%)
Non-bullied	188 (45.2%)

**Table 3:** Association between WBS and the sociodemographic characteristics of the nurses (n = 416).

Factor	Work-Related Score (50) Mean ± SD	Person-Related Score (55) Mean ± SD	Overall WBS Score (105) Mean ± SD
Age group			
20–30 years	20.9 ± 10.7	24.2 ± 11.0	45.2 ± 21.2
31–40 years	23.1 ± 10.1	25.7 ± 10.1	48.8 ± 19.8
>40 years	19.5 ± 9.22	22.3 ± 10.1	41.8 ± 18.9
*H*-test; *p*-value	8.514; 0.014 **	8.518; 0.014 **	8.813; 0.012 **
Sex			
Male	23.8 ± 9.42	26.5 ± 10.0	50.3 ± 18.9
Female	21.5 ± 10.3	24.2 ± 10.5	45.7 ± 20.4
*Z*-test; *p*-value	2.473; 0.013 **	2.207; 0.027 **	2.412; 0.016 **
Marital status			
Unmarried	21.7 ± 10.8	24.6 ± 11.3	46.3 ± 21.7
Married	22.2 ± 9.79	24.9 ± 9.79	47.1 ± 19.2
*Z*-test; *p*-value	0.927; 0.354	0.946; 0.344	1.011; 0.312
Professional rank			
Staff nurse	20.9 ± 10.2	23.9 ± 10.5	44.8 ± 20.3
Charge/Managerial nurse	23.7 ± 10.5	26.2 ± 10.0	49.9 ± 19.6
*Z*-test; *p*-value	3.094; 0.002 **	2.647; 0.008 **	2.978; 0.003 **
Educational level			
Diploma	16.4 ± 7.28	19.3 ± 7.82	35.8 ± 14.7
Bachelor or higher	22.7 ± 10.3	25.4 ± 10.5	48.1 ± 20.3
*Z*-test; *p*-value	4.067; <0.001 **	3.928; <0.001 **	4.128; <0.001 **
Years of experience			
<3 years	22.7 ± 11.1	25.6 ± 11.4	48.3 ± 22.1
3–6 years	25.6 ± 10.1	27.9 ± 9.94	53.6 ± 19.5
>6 years	19.4 ± 9.15	22.4 ± 9.81	41.8 ± 18.6
*H*-test; *p*-value	33.172; <0.001 **	28.722; <0.001 **	32.539; <0.001 **
Work area			
General unit	21.0 ± 10.2	23.9 ± 10.6	45.0 ± 20.5
Specialty unit	22.9 ± 10.0	25.5 ± 10.2	48.4 ± 19.7
*Z*-test; *p*-value	2.171; 0.030 **	1.933; 0.053	2.074; 0.038 **
Hospital			
Alyamama Hospital	17.5 ± 8.41	20.9 ± 8.92	38.4 ± 16.9
Dawadmi Hospital	21.9 ± 9.49	24.6 ± 9.14	46.5 ± 18.3
Prince Mohammad Bin Abdulaziz Hospital	23.9 ± 11.6	27.1 ± 12.3	51.1 ± 23.4
Quayah Hospital	24.5 ± 9.40	26.2 ± 9.30	50.7 ± 18.3
Shaqra Hospital	21.7 ± 9.08	24.0 ± 9.32	45.7 ± 18.0
*H*-test; *p*-value	26.233; <0.001 **	18.684; 0.001 **	22.612; <0.001 **

** Significant at *p* < 0.05 level.

## Data Availability

The data are available upon reasonable request from the corresponding author.
